# Supplementary material for: Controller-driven vector autoregression model for predicting content popularity in programmable named data networking devices
Source: PeerJ Comput Sci. 2024 Feb 8;10:e1854. doi: 10.7717/peerj-cs.1854 (PMC10909199; doi:10.7717/peerj-cs.1854)
Supplement: Supplemental Information 1 [file peerj-cs-10-1854-s001.xz › ndnSIM-code/NFD/docs/named_data_theme/layout.html]

{#
named\_data\_theme/layout.html
~~~~~~~~~~~~~~~~~~~~~~~~~~~~
#}
{%- extends "agogo/layout.html" %}
{% block header %}

# {{ shorttitle|e }}

{% endblock %}
{%- block sidebartoc %}

### {{ \_('Table of Contents') }}

{{ toctree(includehidden=True) }}

### {{ \_('Additional Documentation') }}

- API Documentation (doxygen)
- NFD Wiki

{%- endblock %}
{% block footer %}

This research is partially supported by NSF (Award CNS-1040868).  
  
Licensed under a Creative Commons Attribution 3.0 Unported License except where noted.

{% endblock %}
