# Supplementary material for: Controller-driven vector autoregression model for predicting content popularity in programmable named data networking devices
Source: PeerJ Comput Sci. 2024 Feb 8;10:e1854. doi: 10.7717/peerj-cs.1854 (PMC10909199; doi:10.7717/peerj-cs.1854)
Supplement: Supplemental Information 1 [file peerj-cs-10-1854-s001.xz › ndnSIM-code/NFD/docs/named_data_theme/named_data_header.html]

$projectname: $title
$title

$treeview
$search
$mathjax


# $projectname $projectnumber documentation
