# Supplementary material for: Controller-driven vector autoregression model for predicting content popularity in programmable named data networking devices
Source: PeerJ Comput Sci. 2024 Feb 8;10:e1854. doi: 10.7717/peerj-cs.1854 (PMC10909199; doi:10.7717/peerj-cs.1854)
Supplement: Supplemental Information 1 [file peerj-cs-10-1854-s001.xz › ndnSIM-code/docs/ns3_html_theme/layout.html]

{#
ns-3/layout.html
~~~~~~~~~~~~~~~~~~~
Sphinx layout template for ns-3.
:copyright: Copyright 2012 by ns-3, see AUTHORS.
:license: GPL, see LICENSE for details.
#}
{% extends "basic/layout.html" %}
{% set reldelim1 = ' ' %}
{# set reldelim1 = ' @' #}
{%- block extrahead %}
{%- if theme\_customstylesheet %}
{%- endif %}
{%- if theme\_favicon %}
{%- endif %}
{% endblock %}
{% block header %}

|  |  |  |
| --- | --- | --- |
|  | {{ theme\_projectbrief }} {{ theme\_projectnumber }} | {{ docstitle }} |

{% endblock %}
{% block rootrellink %}- {{ theme\_projectname }}
- {{ theme\_apidocs }}
- Getting started
- Research papers
{#
{{ super() }}
#}
{% endblock %}
{% if theme\_collapsiblesidebar|tobool %}
{% set script\_files = script\_files + ['\_static/sidebar.js'] %}
{% endif %}
