# Supplementary material for: Controller-driven vector autoregression model for predicting content popularity in programmable named data networking devices
Source: PeerJ Comput Sci. 2024 Feb 8;10:e1854. doi: 10.7717/peerj-cs.1854 (PMC10909199; doi:10.7717/peerj-cs.1854)
Supplement: Supplemental Information 1 [file peerj-cs-10-1854-s001.xz › ndnSIM-code/docs/ns3_html_theme/ns3_doxy_header.html]

$projectname: $title
$title

$treeview
$search
$mathjax


|  |  |  |  |
| --- | --- | --- | --- |
|  | NS-3 based Named Data Networking (NDN) simulator ndnSIM 2.5: NDN, CCN, CCNx, content centric networks |
 $searchbox |
 API Documentation |
