# Supplementary material for: Controller-driven vector autoregression model for predicting content popularity in programmable named data networking devices
Source: PeerJ Comput Sci. 2024 Feb 8;10:e1854. doi: 10.7717/peerj-cs.1854 (PMC10909199; doi:10.7717/peerj-cs.1854)
Supplement: Supplemental Information 1 [file peerj-cs-10-1854-s001.xz › ndnSIM-code/docs/source/_static/ndnSIM-structure.pdf]

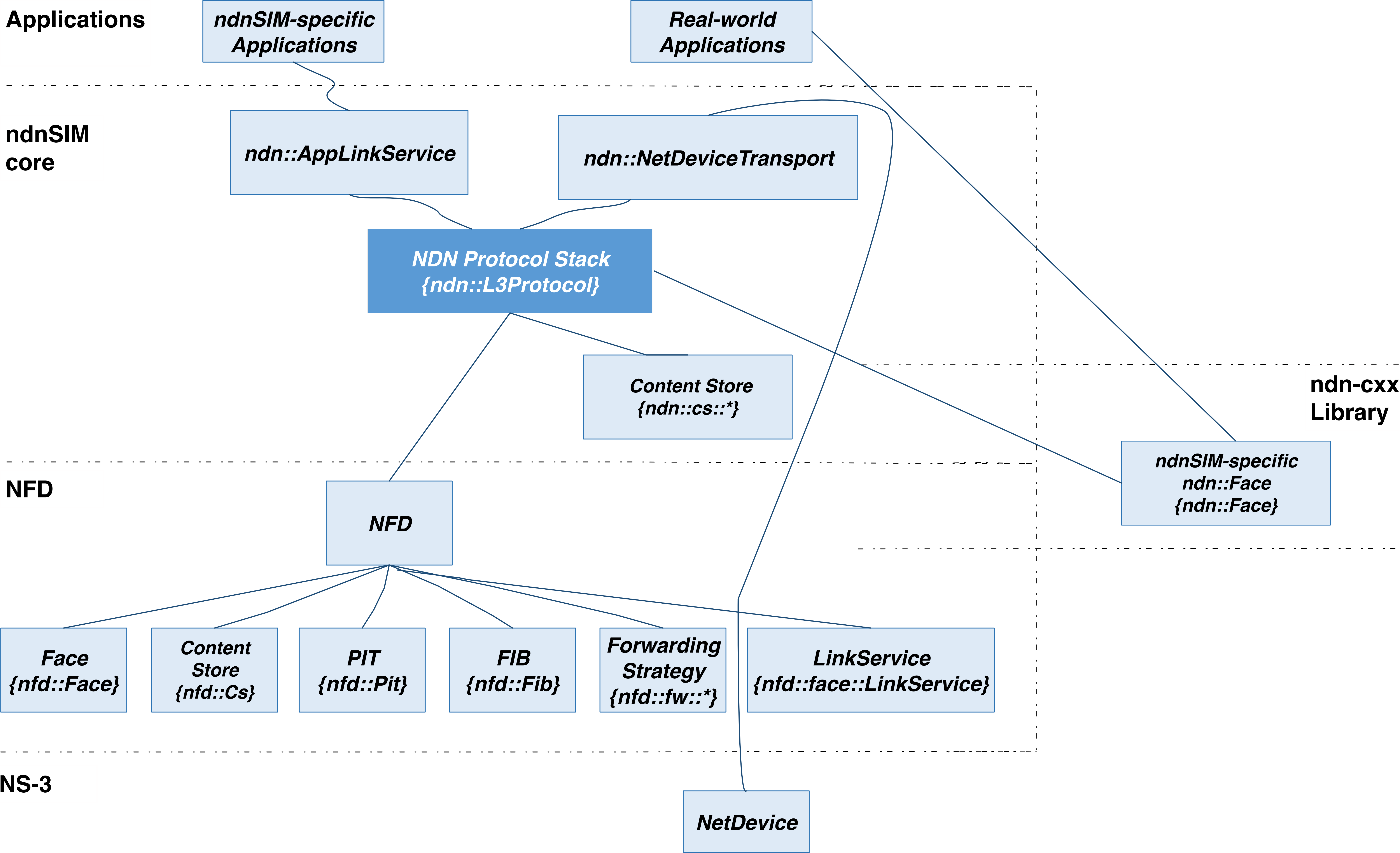
Applications
ndnSIM-specific Applications
Real-world Applications
ndnSIM core
ndn::AppLinkService
ndn::NetDeviceTransport
NDN Protocol Stack {ndn::L3Protocol}
Content Store {ndn::cs::*}
ndn-cxx Library
ndnSIM-specific ndn::Face {ndn::Face}
NFD
NFD
Face {nfd::Face}
Content Store {nfd::Cs}
PIT {nfd::Pit}
FIB {nfd::Fib}
Forwarding Strategy {nfd::fw::*}
LinkService {nfd::face::LinkService}
NS-3
NetDevice
