# Supplementary material for: Controller-driven vector autoregression model for predicting content popularity in programmable named data networking devices
Source: PeerJ Comput Sci. 2024 Feb 8;10:e1854. doi: 10.7717/peerj-cs.1854 (PMC10909199; doi:10.7717/peerj-cs.1854)
Supplement: Supplemental Information 1 [file peerj-cs-10-1854-s001.xz › ndnSIM-code/docs/source/_static/nfd-forwarding-overview.pdf]

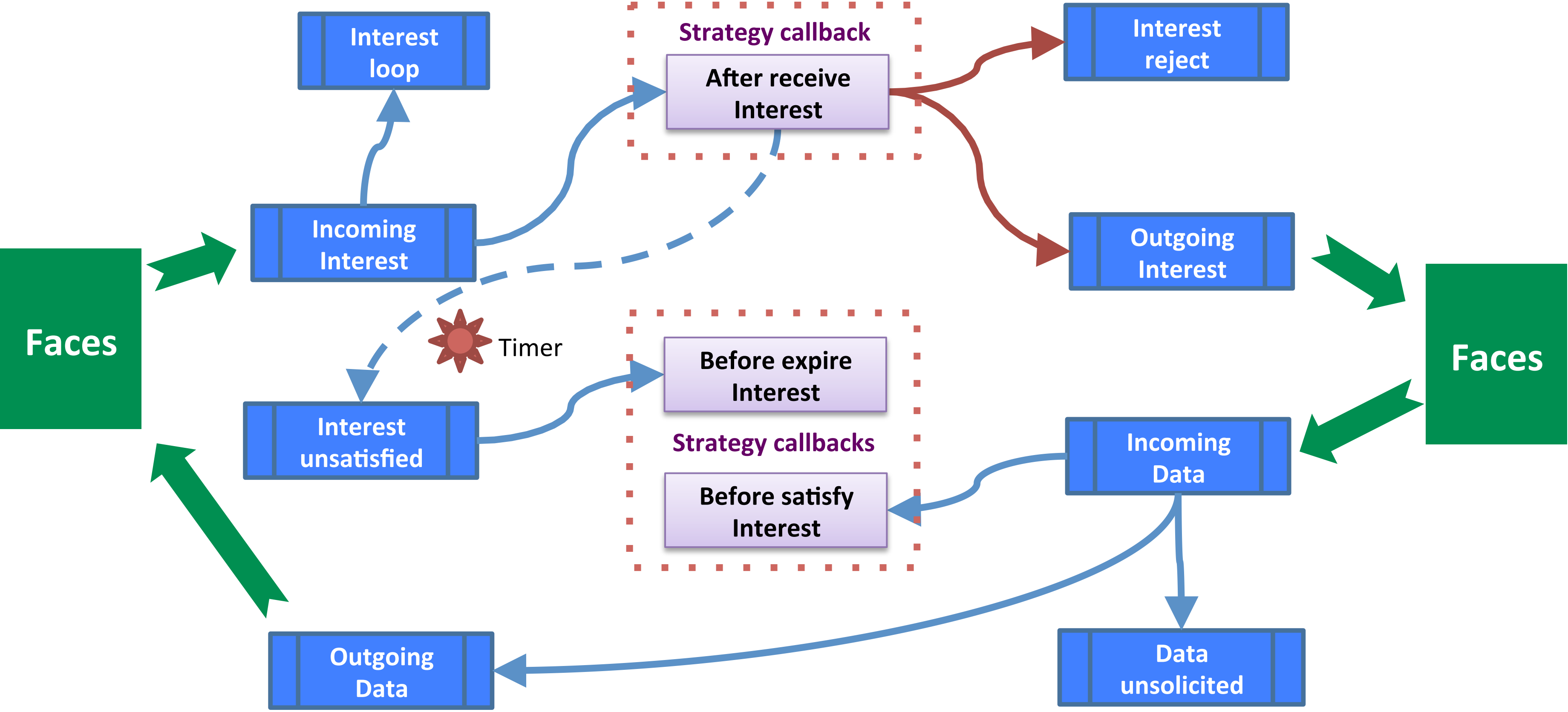

Interest loop
Strategy callback
After receive Interest
Interest reject
Faces
Incoming Interest
Outgoing Interest
Faces
Timer
Before expire Interest
Interest unsatisfied
Strategy callbacks
Incoming Data
Before satisfy Interest
Outgoing Data
Data unsolicited
