# Supplementary material for: Controller-driven vector autoregression model for predicting content popularity in programmable named data networking devices
Source: PeerJ Comput Sci. 2024 Feb 8;10:e1854. doi: 10.7717/peerj-cs.1854 (PMC10909199; doi:10.7717/peerj-cs.1854)
Supplement: Supplemental Information 1 [file peerj-cs-10-1854-s001.xz › ndnSIM-code/docs/source/_templates/indexcontent.html]

{% extends "layout.html" %}
{% set title = \_('ndnSIM Documentation') %}
{% block body %}

# {{ docstitle|e }}

This website includes the ndnSIM documentation and is intended to help the community use the simulator for NDN experimentation. It is not intended to present the principles or the design of the NDN architecture and communication model.
We invite you to join our mailing list to see and participate in discussions about ndnSIM implementation and simulations in general (mailing list archives).

{% block tables %}

|  |  |
| --- | --- |
| Getting Started  Introduction  what is ndnSIM, how to get support, and what is in the package  Downloading and Compiling ndnSIM  requirements, downloading, compiling, simulating  FAQs  frequently asked questions (with answers!)  Simulation examples  basic tutorial examples  Best practises  best practises on using ndnSIM | Advanced Topics  ndnSIM API documentation  generated documentation of ndnSIM API  Parallelize simulations using OpenMPI  how to use OpenMPI to parallelize execution of simulation scenarios  Simulating real applications  guide on how to simulate real application that are written against ndn-cxx library |
| ndnSIM tutorial  ndnSIM helpers  very important components of ndnSIM for writing simulation scenarios  Content Store  several different build-in content store implementations: LRU, LFU, and others  Forwarding Strategies  experimenting with different build-in and custom Interest/Data forwarding strategies  ndnSIM applications  reference applications for network-leverl evaluations  Obtaining metrics  obtaining and visualizing metrics from simulation runs | ndnSIM Versions  ndnSIM 2.9  the latest version  ndnSIM 2.8  ndnSIM 2.7  ndnSIM 2.6  ndnSIM 2.5  ndnSIM 2.4  ndnSIM 2.3  ndnSIM 2.2  ndnSIM 2.1  ndnSIM 2.0  ndnSIM 1.0 |
| Publications  ndnSIM-related publications  general papers about ndnSIM, research papers that use ndnSIM  If you are referring to ndnSIM in a published work, please cite the following papers, not just the ndnSIM website. Thank you!  - **For ndnSIM >= 2.0:**  - S. Mastorakis, A. Afanasyev, and L. Zhang, "On the Evolution of ndnSIM: an Open-Source Simulator for NDN Experimentation," ACM SIGCOMM Computer Communication Review (CCR), July 2017 (PDF) (BibTex) - S. Mastorakis, A. Afanasyev, I. Moiseenko, and L. Zhang, “ndnSIM 2: An updated NDN simulator for NS-3,” NDN, Technical Report NDN-0028, Revision 2, 2016 (PDF) (BibTex)  - **For ndnSIM 1.0:**  - A. Afanasyev, I. Moiseenko, and L. Zhang, “ndnSIM: NDN simulator for NS-3,” NDN, Technical Report NDN-0005, 2012 (PDF) (BibTex) | Miscellaneous  Release Notes  new features, changes, bug fixes, deprecations, and removals  Reporting bugs  ndnSIM Redmine, GitHub pull requests  ndnSIM mailing list  mailing list archives  Community Contributions  very much appreciated contributions of the ndnSIM user community  ndnSIM Team  Copyright and License |

{% endblock %}
{% endblock %}
